# Dental Service and Resource Needs during COVID-19 among Underserved Populations

**DOI:** 10.1177/23800844221083965

**Published:** 2022-03-17

**Authors:** V. Johnson, M. Brondani, H. von Bergmann, S. Grossman, L. Donnelly

**Affiliations:** 1 Faculty of Dentistry, University of British Columbia, Vancouver, BC, Canada; 2 Centre for Community Engaged Learning, University of British Columbia, Vancouver, BC, Canada

**Keywords:** access to care, pandemic, qualitative, vulnerable populations, dental care delivery, oral health care

## Abstract

**Introduction:**

In response to the coronavirus disease 2019 (COVID-19) outbreak, dental services in British Columbia, Canada, were restricted to urgent and emergency cases between March 16 and May 18, 2020. It is unclear how the curtailment of oral health services has affected underserved populations who already often have limited access to dental care due to cost, fear, stigma, and discrimination.

**Objectives:**

To explore the experiences of underserved populations and their community organizations when accessing oral health services and information in British Columbia and identify their coping mechanisms employed during the curtailment of oral health care services.

**Methods:**

Semistructured, remote interviews were conducted with 13 staff and 18 members from 6 community-based organizations. These organizations serve men and women with a history of incarceration and/or experiencing poverty and homelessness, persons living with human immunodeficiency virus/AIDS, adults living with mental illness, and older adults in long-term care facilities. The interviews were audio-recorded, transcribed verbatim, and coded for emerging themes using NVivo 12 software. Thematic analysis was performed.

**Results:**

The pandemic raised concerns and hesitancy among underserved populations and further reduced access to care. In turn, those with unmet dental needs resorted to coping mechanisms, including turning to community support or medical services, self-management of dental issues, and not dealing with dental issues altogether. Community organizers and members outlined needed resources such as assistance navigating the dental care system, having a contact for dental-related questions, and member preparation for dental service changes, while emphasizing the importance of positive relationships with dental providers.

**Conclusion:**

Underserved populations who already face barriers to oral health care services experienced increased difficulty in addressing their oral health needs and concerns during the beginning of the COVID-19 pandemic. Strategies aimed at reaching out to this population and those who support them are needed to help mitigate negative coping strategies and increased oral health disparities.

**Knowledge Transfer Statement:**

This study depicts ways of addressing unmet oral health–related issues during the COVID-19 pandemic for underserved populations and their community organizations with policy implications as well as practical strategies.

## Introduction

In Canada, dental care is primarily delivered through the private sector and effectively supports the oral health of the majority of Canadians, but inequalities in oral health and inequitable access to dental services disproportionally affect vulnerable and marginalized populations (Canadian Academy of Health Sciences 2014). Approximately 6% of all dental care expenditures in Canada are publicly funded (Canadian Academy of Health Sciences 2014), and only 5.5% of all Canadians have public dental benefits (Health Canada 2010) while 35.4% do not have dental insurance (Statistics Canada 2019). Although public dental programs are available for specific target underserved populations, including children living in low-income families, social assistance recipients, people with disabilities, and Aboriginal peoples, they are often limited and insufficient in meeting their needs and other underserved groups, such as low-income adults and older adults, are excluded (Bedos et al. 2003; Wallace and Macentee 2012; Canadian Academy of Health Sciences 2014). In addition, those eligible for public dental benefits often face complicated insurance-related barriers to accessing dental care such as dental providers’ aversion to treating publicly funded patients due to dental providers’ being dissatisfied with the public plans (Quiñonez et al. 2009).

Vulnerable populations are defined as having at least 1 interrelated dimension of vulnerability such as reduced individual capacities, lack of support networks, or their community lacking or having barriers to access essential services (Mechanic and Tanner 2007). One of these services includes dental care. In Canada, these underserved populations who struggle to access dental services include people with low incomes, children in low-income families, people without dental insurance, older adults residing in institutions or with low incomes, Aboriginal peoples, refugees and newcomers, people with disabilities, and people living in rural regions (Canadian Academy of Health Sciences 2014). People living with human immunodeficiency virus (HIV) or AIDS in particular have a stigmatizing, chronic condition that may lead to disability; hence, they are also identified as being vulnerable. Furthermore, the behavior model for vulnerable populations outlines factors that inhibit access to care such as mental health, substance use, competing needs, and incarceration history due to their social structure characteristics (Gelberg et al. 2000). All these above underserved populations continue to face multiple barriers to accessing dental care such as cost, fear and dental phobia, denial of care, stigma, and discrimination that contribute to disproportionately higher rates of untreated dental caries, periodontal diseases, missing teeth, and oral pain, as well as greater dental care needs (Canadian Academy of Health Sciences 2014; Brondani et al. 2017; Slack-Smith et al. 2017; Donnelly et al. 2019; Jessani et al. 2020). Substantial research supports that low-income Canadians face financial barriers to accessing dental care that contribute to lower dental care utilization as well as poorer oral health outcomes (Health Canada 2010; Canadian Academy of Health Sciences 2014). For people with severe mental illness, factors contributing to oral disease include amotivation, poor oral self-care, dental phobia, dental costs, difficulty accessing dental care, adverse effects of psychotic medications such as oral dryness, and stigma from dental professionals (Brondani et al. 2017; Slack-Smith et al. 2017). Barriers to accessing dental care among formerly incarcerated people include indifference toward oral health, stigma of incarceration, negative previous dental experiences, restrictions from parole or community supervision, and institutional conditioning (Donnelly et al. 2019). Frequently reported challenges that impede access to dental care for people living with HIV/AIDS include cost, dental anxiety and fear, transportation issues, and dental provider discrimination and stigma (Jessani et al. 2020). Similarly, older adults face financial barriers, dental anxiety, ageism from dental professionals, reduced expectations of having their dental needs addressed, and transportation challenges (Yao and MacEntee 2014). Consequently, these adults with vulnerabilities experience greater oral health inequities when compared to the general Canadian population (Wallace et al. 2015).

The coronavirus disease 2019 (COVID-19) global pandemic caused by the severe acute respiratory syndrome coronavirus 2 (SARS-CoV-2) has raised concern among experts over oral health inequalities becoming exacerbated (Brian and Weintraub 2020) as dental services were restricted to urgent and emergent during the first wave of infections (Brondani and Donnelly 2021). Vulnerable and marginalized populations with already limited access to needed dental services (Canadian Academy of Health Sciences 2014; Brondani et al. 2017; Slack-Smith et al. 2017; Donnelly et al. 2019; Jessani et al. 2020) were at risk of being further affected by the curtailment of oral health care services. Previous studies have found that when access to oral health care services is limited, individuals may rely on hospital emergency rooms and substance use to cope with or address their oral problems (Brondani and Ahmad 2017). In the United States, dental service utilization among insured patients decreased during the first wave of SARS-CoV-2 infections and reached the lowest point during the week of April 6 when there was a 94.5% reduction in service utilization compared to the previous year (Choi et al. 2021). Kranz et al. (2021) reported 66% fewer weekly dental visits in the week of April 12, 2020, across the United States. Similarly, a poll of US dentists revealed that in the week of April 20, 2020, 86.0% of respondents reported that their total patient volume was significantly reduced to less than 5% compared to their typical patient volume before the COVID-19 pandemic (Health Policy Institute 2021). After restrictions were eased, dental service utilization began rebounding in the private sector but remained lower within the publicly insured population (Choi et al. 2021; Kranz et al. 2021). The full impact of the COVID-19 pandemic on oral health service utilization by underserved populations remains unexplored in British Columbia.

This study aimed to: 1) explore the experiences of underserved populations and their community organizations when accessing oral health services and information in British Columbia; and 2) identify their coping mechanisms employed during the curtailment of oral health care services. These 2 aims are addressed under the following research question: how did the COVID-19 pandemic affect dental services access and needs for underserved populations in British Columbia, Canada, during the first 6 mo of the pandemic?

## Methods

A transformative research paradigm (Mertens 2009) under a social justice framework was employed in this qualitative study that involved those who benefited from the research since its inception. This framework provides guidance for research projects collaborating with historically marginalized populations in methodological decision-making and conducting research to promote social justice. Over the past decade, the Faculty of Dentistry at the University of British Columbia has been collaborating with 6 community-based organizations that serve a variety of underserved members of our society to increase access to oral health care professionals within their communities. These populations historically experience structural vulnerabilities that result in reduced access to dental care and higher levels of oral disease (Canadian Academy of Health Sciences 2014). These 6 organizations serve men and women with a history of incarceration and/or experiencing poverty and homelessness, persons living with HIV/AIDS, adults living with mental illness, and older adults in long-term care facilities. In turn, community-based staff and organizers from the 6 partnering organizations aided in designing the interview questions by offering suggestions of questions that would be important to address, providing feedback to ensure the wording was appropriate, and partaking in the initial interviews as participants where they introduced concepts that were incorporated into the interview scripts for their members to later elaborate on. The staff collaborators also offered recruitment recommendations for potential community members who may be interested in participating in the study, although they did not engage in any members’ individual interviews. The collaborators were community partners who we have worked with for many years and who were in administration of service positions for their organizations. Potential participants were recruited to partake in individual interviews to gain insight into their experiences accessing oral health care services and information, as well as their dental needs during the temporary service curtailment and the COVID-19 pandemic. All participants provided informed consent verbally, and 18 participants also provided written consent. Inclusion criteria were those older than 19 y of age and able to communicate in English. Recruitment posters with information on the study details were distributed and advertised at each of the 6 participating community-based organizations. We aimed at purposefully selecting participants with a diverse representation of age, gender, and life experience within and between the different organizations. Although the participants were all affiliated with organizations serving specific targeted underserved populations, there was considerable heterogeneity among the participants. Approval for this study was obtained from the UBC Behavioural Research Ethics Board (BREB #H20-02070-A001).

### Interviews

In-depth, semistructured, at-a-distance individual interviews were conducted with community-based staff or organizers from each community organization and with their members over the phone or via Zoom between July and December 2020. Interview guide questions were framed to identify how community organizers and members were addressing oral health issues as they arise, what barriers and facilitators they were experiencing in accessing needed services, how they were coping with potentially reduced access, how they were coping with any oral problems, what resources they were relying on, and what types of services they would find essential and necessary now and in the future. Two authors (VJ, LD) conducted 7 interviews to calibrate interview conduct, and then the first author (VJ) conducted the remainder of the 24 interviews. Each interview lasted between 30 and 60 min and was audio-recorded. Participants were offered a $25 honorarium for their time devoted to the study.

### Data Analysis

All interview audio-recordings were transcribed verbatim and coded for emerging themes using NVivo 12 software (QSR International) and thematic analysis. Participants’ names and other identifiers were anonymized. Transcripts were reviewed multiple times by the first author (VJ) for familiarization, and then patterns of meaning within the transcripts were identified and interpreted through coding. Coding is an inductive process whereby a code in the form of a word or phrase is assigned to an excerpt of a transcript that describes the essence of what is being said. Two authors (VJ, LD) individually coded 2 of the same interviews, then compared codes for consistency and reached consensus. The first author proceeded to code 10 transcripts, discussed the developing list of codes with the other two authors (MB, HvB), and coded the remaining 19 transcripts. The analysis led to a total of 121 codes, and then similar codes were grouped into 21 categories that describe an aspect of a shared particular occurrence. Examining the similarities and finding relations between categories gave rise to 4 overarching themes and 12 subthemes that were organized sequentially to explain the process in participant experiences. The researchers employed reflexivity throughout data collection, data analysis, and reporting by continually recognizing their socioeconomic positions that enabled them to avoid personal experiences with discrimination and access to dental care issues, as well as being conscious of the potential power imbalances between researchers and members, researchers and staff, and staff and members. To address these potential biases and power imbalances, the researchers built rapport with the participants and performed active listening during interviews, as well as internally reflected on personal assumptions and values throughout data analysis. To improve trustworthiness and achieve rigor in this study, interviews were conducted until data saturation was reached when no new information was being discovered. After the initial data analysis, the study results were presented to each community organization, and all organizations were invited to provide feedback on the findings. Moreover, all participants were provided with the opportunity to review, clarify, and add further insight to their own personal transcripts and the initial data analysis, although only 1 participant provided feedback on the analysis of their own transcript.

## Results

A total of 31 interviews with 13 community-based staff or organizers and 18 members were conducted, resulting in 18 h of audio recordings and 250 pages of written transcripts that were analyzed thematically. A summary of the participant demographic characteristics, including gender, staff or member position at the community organization, and the organization’s target population, is described in the Table. Four main themes with nine subthemes emerged from the analysis: concerns (COVID-19 anxiety, COVID-19 safety protocols), access to services (dental service needs, disruptions of care), coping (accessing community support or medical services, self-management of dental issues, not dealing with dental issues), and needs and suggested solutions (information, resources).

**Table.** Demographics.

Participant No.	Gender	Staff or Member	Organization No.	Staff Position
1	Female	Staff	1	Service coordinator
2	Female	Staff	2	Service coordinator
3	Male	Staff	1	Outreach worker
4	Male	Staff	3	Clinician
5	Male	Staff	4	Service coordinator
6	Male	Staff	5	Administrator
7	Female	Staff	4	Supervisor of resources
8	Female	Staff	5	Administration
9	Female	Staff	1	Outreach worker
10	Male	Staff	6	Outreach worker
11	Male	Staff	6	Outreach worker
12	Female	Staff	4	Community integration specialist
13	Female	Staff	5	Service coordinator
14	Male	Member	1	
15	Male	Member	6	
16	Female	Member	2	
17	Female	Member	2	
18	Female	Member	2	
19	Female	Member	6	
20	Male	Member	6	
21	Male	Member	5	
22	Female	Member	5	
23	Male	Member	5	
24	Male	Member	5	
25	Male	Member	5	
26	Male	Member	5	
27	Male	Member	1	
28	Male	Member	6	
29	Male	Member	1	
30	Male	Member	1	
31	Male	Member	6	

Organization 1 serves individuals experiencing homelessness, substance use, mental health challenges, developmental disabilities, and/or criminal justice involvement. Organization 2 serves women and children at risk, involved in, or affected by the justice system.* Organization 3 serves older adults in long-term care facilities. Organization 4 serves people experiencing poverty, homelessness, and addiction. Organization 5 serves people living with mental illness. Organization 6 serves people living with human immunodeficiency virus/AIDS.* (*Members from these organizations also experience poverty, addiction, mental illness, and homelessness.)

## Theme: Concerns

### COVID-19 Anxiety

The COVID-19 pandemic had significantly affected people’s daily lives as public health orders were introduced to reduce the spread of the virus. These disruptions to regular life, the uncertainty amid the novelty of the pandemic, and the potential threat of the virus induced feelings of fear and stress as voiced by the participants:

The first few months . . . we were kind of like very intimidated by the whole thing. And I was scared. I was washing my hands so often. . . . And I was afraid of going outside. . . . And I saw on the news people were being bagged up in body bags. . . . Six hundred people at a day dying. . . . I was crying inside but I was like just too freaked out to really even cope with that. (Male member, organization 5)

The fear of contracting COVID-19 led some members to avoid or hesitate to access various types of services in order to reduce their risk. As 1 staff participant stated, “The amount of women accessing our [community] service was reduced as well and just limited. I think in light of COVID there may be more of a hesitation to access services. Not just dental but all services” (female staff, organization 2).

Members with underlying health issues, such as those with respiratory conditions or HIV/AIDS, were particularly more cautious with reducing possible exposure to COVID-19 since they were considered to be at risk of greater disease severity. One male member from organization 6 told us,

I was kind of worried about going into the dental office . . . because I have underlying issues that makes me more susceptible to COVID so . . . I was worried about catching COVID.

A female staff from organization 1 similarly expressed concern for the health of their members who were medically compromised:

[My member] is such high respiratory risk that she’s not going anywhere . . . she normally travels by handy dart but they’re sort of deeming that still too high risk for her so she’s not going to receive any dental care for the next little while. . . . Because the population that we work with . . . [have] many underlying health conditions and also sometimes addictions . . . I feel like if anybody got sick with this virus unfortunately they would be very sick and they wouldn’t be asymptomatic.

### COVID-19 Safety Protocols

The new safety protocols implemented to prevent and control the transmission of COVID-19 in dental offices include increased screening and enhanced personal protective equipment (PPE), although participants had mixed perspectives on the changes.

Concerns about the sufficiency of dental offices’ COVID-19 safety protocols were raised as some participants did not know the level of safety or risk of transmission when accessing dental services. As 1 member indicated, “I don’t know what they have in place right so that makes me very anxious. Was someone sitting in the waiting room, is it contaminated, has it been cleaned, how often does it get cleaned? . . . All that not knowing is a big concern for me” (male member, organization 6).

In contrast, other members trusted that dental offices would adhere to safety protocols and standards in order to provide care safely:

As a patient I have [no concerns], because I know that the Ministry [of Health] and the dentists and the dental review boards will be the ones that are looking and scrutinizing all the care. . . . I know that if a dentist will look at me that that dentist feels comfortable in the environment that he’s in. . . . So it’s kind of like I’m relying on everyone else to do their job. (Male member, organization 6)

Approximately half the staff participants similarly felt comfortable resuming dental services during the pandemic either themselves or for their members since they trusted that the dental professionals would follow appropriate safety protocols. This increased confidence in the safety protocols among staff participants may be attributed to their greater understanding and familiarity with COVID-19 regulations given their own organizations’ protocols:

I don’t have concerns because I understand what you’re doing . . . and because we had to initiate some of these [regulations], we’ve been living and breathing COVID for the last four months. So we’re quite familiar with what COVID is all about, how it’s transmitted, the steps that we have to take ensuring our safety and we’ve been successful here and we’ve gone for four months without an issue here. (Male staff, organization 5)

The new COVID-19 safety protocols also included raised minimum requirements for PPE worn by dental professionals to improve their safety, including a level 3 surgical mask or higher, gown, and face shield or goggles. However, the enhanced PPE may have caused communication difficulties between members and dental professionals, depersonalized interactions, or triggered increased stress among members with negative past experiences in medical environments. As 1 staff participant indicated,

If [members] don’t know what you look like and you’re just wearing a mask and that’s what you see it’s quite intimidating. . . . It’s just hard I think for them especially if you’re uncomfortable already in a medical setting. (Female staff, organization 4)

Nonetheless, most members and staff were well adapted and not concerned with the enhanced PPE worn by dental professionals, which reassured safety: “I think [enhanced PPE] would probably make you feel less worried about it if I saw that. Because then I know they would be doing steps to keep themselves safe and myself safe” (female member, organization 2).

Over time, the fear of and anxiety for the threat of COVID-19 were decreased by the new regulations and enhanced PPE, as reflected by a female staff: “At the beginning [members] were always asking me for masks because their staff [were] wearing them but they’re a measure to keep them safe. . . . And I think a lot of the anxiety and maybe paranoia around it [like] why do you need to wear them and we don’t has lessened” (female staff, organization 5).

## Theme: Access to Services

Many medical, dental, and community service providers implemented changes to their regular practices to reduce the risk of transmission of COVID-19. The new COVID-19–related changes and restrictions to services led for the most part to reduced access to services and introduced new disruptions to care, such as delays and denial of care.

### Dental Service Needs

Most participants agreed that the dental needs did not change during the pandemic. As 1 male member from organization 6 remarked, “The [dental] needs are the same,” but access to dental services has changed: “it’s just harder to access now” (male staff, organization 1). Many members wanted to continue the same routine dental services as prior to the pandemic or complete previously planned treatments: “I had the cleanings halted. . . . I was supposed to go in before the pandemic but then everything shut down after the pandemic” (male member, organization 6). However, staff participants also emphasized that existing dental problems could progress and worsen during this time when only essential dental services were being provided, potentially increasing the needs:

I would say [dental needs] actually got amplified because there were many people that had processes in place up until the shutdown and then all the sudden all the offices were just closed. . . . I mean I know the emergency [dental care] was still taking place but there’s lots of people kind of caught in that little area where it’s not quite enough to be considered an emergency. So you know maybe they’ve had a temporary [filling] now on four or five months rather than you know a few weeks and how long is that going to last. (Male staff, organization 6)

### Disruptions of Care

Half of the member participants and three-quarters of the staff participants reported having their dental appointments delayed, cancelled, or rescheduled, which the majority attributed to dental offices being closed or limiting service earlier on. As 1 male staff from organization 6 commented,

Everything’s been put on hold because dental offices were closed except those few that remained open for emergency services. I had my own appointments canceled around me because of the closures that were ordered and I’ve yet to get back to seeing anyone yet.

Participants also discussed the issue of being an essential worker and accessing care: “Frequently you’re not getting [dental services] because you’re an essential services worker and have a higher risk factor of being exposed to COVID” (male staff, organization 6). This experience may indicate dental offices’ reluctance to provide care to those who were at high risk of exposure to COVID-19 in order to minimize the risk of transmission in their offices.

Not being a patient of record was cited as another reason for not being able to access needed care, as we were told that “[members] would phone around and other dentists would say ‘sorry you’re not one of our regular patients so . . . we can’t slip you in as an emergency.’ So I have had people that are suffering with the pain” (female staff, organization 4). Although such reasoning left members without a regular dental home with fewer options during the curtailment period, over one-third of the member participants and the majority of staff participants informed us that they were able to access the services as restrictions eased off. As 1 male member from organization 5 said, “I went to the dentist . . . as soon as they started practicing again.”

## Theme: Coping

Members who did struggle to access needed oral health care services described various coping mechanisms to deal with their dental issues. These coping mechanisms included accessing community support, primary care, or urgent care services, self-management of dental issues, and not dealing with dental issues altogether.

### Accessing Community Support or Medical Services

When experiencing dental pain and unsuccessfully accessing dental services, some members turned to community staff for support, urgent care, and/or primary care services to address their dental problems. However, community staff similarly struggled to support members with immediate dental needs and could only provide them with reduced-cost dental clinics’ contact information. In addition, primary and urgent care offered services limited to analgesic or antibiotic medication as they could not treat the source of the dental pain:

We have had a few members successfully receive emergency dental care but even the emergency dental care was like OK I’ll see you in eleven days. This guy is screaming in pain . . . so we ended up even bringing some dental emergencies to the urgent care center . . . for dental stuff because the pain was so bad. . . . We tried to call that member’s primary care physician [who] said like “Well I can’t prescribe you antibiotics over the phone because I haven’t seen you in over a year” . . . and so then you know that’s a phone thrown at a wall and a door slammed in the face. . . . So then that was when my coworker took that member to the urgent care and he was prescribed antibiotics. . . . In both cases that I knew of it took about two weeks of pain, which is horrible. (Female staff, organization 1)

### Self-management of Dental Issues

Members experiencing dental pain without access to dental services during the pandemic also described their reliance on analgesic or antibiotic medications, illicit drugs, and oral products to cope with the suffering:

[I took] like a tooth solution . . . it cleans your mouth and it numbs your gum like kill the nerves or whatever and heal the pain. . . . [And I took] Advil and Tylenol like painkillers because you know when you have a toothache you feel pain yeah so basically I took that and it helps. (Male member, organization 1)

For others with a history of substance use or addiction, the potential to revert to using substances as a coping mechanism for stress or pain during the pandemic was apparent:

Even just like stress coping with all these [COVID-19] rules . . . a lot of people that we work with just have coping tools that they lost . . . or they’re not conducive to living a life that they would like to live. Also like I mean drugs are a great coping tool and especially because they’re immediate but then it contributes to the problem so like how do we move towards healthier coping skills in terms of stress . . . [for] people dealing with like addiction particularly that’s really [a] coping tool. (Female staff, organization 1)

Unfortunately, 1 staff participant noted an increase in members expressing that they would perform self-extractions to resolve their dental pain during the pandemic:

It’s so painful . . . they do crazy things. I’ve had members pulling out their own teeth because they figured that’s what’s going to help. (Female staff, organization 4)

### Not Dealing with Dental Issues Altogether

Some members also indicated that they avoided dealing with dental problems, especially if they were no longer being the source of pain:

[My dental pain] started again like after the pandemic started. I was like Oh my God. I’m in so much pain and I couldn’t go to the dentist but it went away. . . . I didn’t go to the dentist again after that . . . I’m like whatever it’s going to stop the pain it’s going to stop the pain it’s going to stop and it actually did stop like it wasn’t painful anymore like there’s no more holes in my mouth. . . . I found it so odd too, it just doesn’t hurt anymore. I’m like what’s going on with my body, but it’s good it wasn’t hurting anymore it went away on its own. (Female member, organization 5)

While the above coping mechanisms may temporarily help people manage pain, some may also lead to harm, such as the use of illicit substances. In turn, participants identified resources and services that could help them throughout the COVID-19 pandemic and beyond.

## Theme: Needs and Suggested Solutions

Various resources, information, and services were suggested to be necessary and essential by the members and staff.

### Information

Providing members and staff with appropriate and current information about the pandemic, available dental services, and ways to prevent dental problems would help address expectations and dental concerns, as well as ease anxiety.

After the temporary suspension of nonessential dental services, participants discussed the need for reconnecting members with services, so that routine dental services could be reestablished and new treatment could be initiated:

[Members] really need the help to reconnect with services. Because [dental needs are] not always prioritized. . . . Information about who is already open, who is more available and likely to do that kind of stuff could be helpful. (Male staff, organization 1)

Another suggestion was to prepare members for the COVID-19 safety protocols in dental offices. When discussing preparation for members, 1 male member from organization 6 discussed how “being shown at the appointment or prior to the appointment what the new implementations are” would increase their comfort with resuming dental care.

In addition, participants recommended informing members about the safety of receiving dental care, as mentioned by a female staff from organization 1:

I think just like access, how to access resources during like while keeping people safe. . . . Information around . . . the risk of transmission and how could it be transmitted through a dental clinic . . . because I think there’s a lot of fear in general. . . . It also helps [staff] fall back on so if people have anxiety it’s like “but these are the facts” . . . rather than letting our brains do the fear mongering.

### Resources

In addition to information, participants requested necessary resources to support underserved populations throughout the pandemic, including reduced-cost or low-barrier dental clinics when nonessential dental services were temporarily suspended:

I think having, especially right now with COVID, again a list of places that they can go to if they need further dental care. Free dental care so like referrals to dentists nearby, not too far. (Female staff, organization 4)

Participants also discussed how having a contact point of information to answer dental-related questions would be helpful:

Being given the tools to be able to refer somebody, to ask the questions they have, to be given the tools on access. . . . I think having being able to say “Well actually there’s a place you can phone that can give you some basic answers. They’re available to you.” I think that would mean a lot . . . and I think it would change people’s opinions about accessing dental as well. Knowing that somebody actually would listen to them and give them an answer. . . . Having again that dental line or having somebody to contact that’s huge. (Female staff, organization 4)

Finally, many participants emphasized the importance of dental providers building rapport and supporting positive, cooperative relationships with their members from underserved populations. This would likely improve member comfort, particularly during the pandemic when greater anxiety was experienced:

Just the whole “How are you? How’s your day been?” That level of empathy or sympathy, however it needs to be expressed is something that I think is just definitely necessary for all of us during this [COVID-19] time period. When I’m going into a dental appointment and I see that they have their hazmat suit on and I’m ready to get my dental care done, just the typical before we even start, “How’s your day been” or “Hi how are you” that moment where we can remember we’re still human before we even start anything, I think is definitely [important] for me personally. (Male member, organization 6)

## Discussion

As dental services were temporarily curtailed in British Columbia at the beginning of the COVID-19 pandemic, we aimed to explore the experiences of underserved populations and their community organizations when accessing oral health services and information in British Columbia and to identify the coping mechanisms they employed to deal with oral health care problems and what resources they required during that period. As we posed our research question, “How did the COVID-19 pandemic affect dental services access and needs for underserved populations in British Columbia, Canada, during the first 6 mo of the pandemic?” we found that the pandemic raised concerns and hesitancy among these populations and further reduced access to care. In turn, those with unmet dental needs resorted to coping mechanisms, including substance use, turning to community support, urgent care, or primary care, and persevering with dental pain. Community organizers and members outlined needed resources such as assistance navigating the dental care system, having a contact for dental-related questions, and member preparation for dental service changes while emphasizing the importance of positive relationships with dental providers.

As the COVID-19 pandemic brought significant disruptions to daily lives, many participants felt heightened anxiety that led some to avoid accessing services, particularly those with increased susceptibility to the COVID-19 virus. Increased anxiety and psychological distress related to the COVID-19 pandemic among the general public have been well documented (Salari et al. 2020). Moreover, Mazza et al. (2020) also found that having a history of medical problems has also been associated with greater levels of depression and anxiety during the pandemic, possibly due to feeling more vulnerable to contracting SARS-CoV-2. The fear of contracting the virus led participants in this study to avoid or hesitate to access various services, including dental and medical services similar to other studies (Guo et al. 2020; Hartnett et al. 2020; Gomez et al. 2021; Walter et al. 2021; Ibrahim et al. 2021; Moharrami et al. 2021). Those who did try to access dental services during this time might have experienced delayed diagnosis, untreated oral diseases, and compromised health (Wall et al. 2012). Although there have been very few documented cases of COVID-19 infection during dental procedures (Froum and Froum 2020; Levit and Levit 2021), the theoretical association between COVID-19 infection and the production of aerosols indicates a risk of occupational transmission (Banakar et al. 2020; Lo Giudice 2020). Overall, it is imperative to address public concern about the safety of accessing dental services and the importance of the protocols during the pandemic to facilitate patient return to services.

Participants, including those with dental pain, might have been denied dental care due to office standards or not being a patient of record, which may have increased oral infections, periodontal problems, and dental caries during the pandemic (Moharrami et al. 2021). Walter et al. (2021) found that the pandemic led to temporary decreases in both urgent and nonemergency patient visits and, like our participants, reinforced the need of maintaining accessibility to dental services for pain-driven urgent dental care. Fortunately, the restrictions on nonessential dental services in British Columbia were implemented only for a few months, and most participants were eventually able to access dental services again.

Participants with dental problems who were unable to access to services resorted to coping mechanisms, including accessing community support or medical services, not dealing with the issue, and self-management of dental issues, as reported by others even before the COVID-19 pandemic, although these strategies do not resolve the underlying dental issue (Brondani and Ahmad 2017). The fact that some members resorted to performing their own extractions is not new and has been documented previously when access to dental care is unavailable and oral pain is significant (Bedos et al. 2003).

A number of resources, information, and services were deemed necessary by participants during the pandemic in our study. However, there is limited evidence beyond commentaries and opinion pieces that explore how to better support underserved populations with access to dental services during the pandemic. Brian and Weintraub (2020) as well as Dziedzic et al. (2020) echoed the importance of improving communication regarding patient safety as they may feel anxious and hesitate to resume dental services. Cotrin et al. (2020) also added how dental providers must follow safety protocols to improve patient confidence and the patient–provider relationship. Messaging to support patient return to regular dental care should include the significance of maintaining oral health and its impact on systemic health (Brian and Weintraub 2020). In addition, Dziedzic et al. (2020) highlight the need for dental providers to show genuine empathy to patients, especially given the difficulties many faced during the pandemic.

Having a dental professional to turn to during times such as this was discussed by many of our participants, who suggested improving access to needed information in a safe manner, likely via telehealth, as recently presented by Brondani and Donnelly (2021) when introducing a preparedness model of oral health care. Virtual platforms such as teledentistry have been proposed to address access disparities since it has the potential to increase the reach of dental providers to typically underserved low-socioeconomic status, disadvantaged groups and rural populations (Estai et al. 2018; Irving et al. 2018). Teledentistry strategies can include remote screening, caries detection, diagnosis, consultation, triage, treatment planning, and education (Estai et al. 2018; Irving et al. 2018). During periods when dental offices are closed and people mainly stay at home, information and communication can be delivered through teledentistry to reduce the burden on hospital emergency rooms (Brian and Weintraub 2020). The need of virtual resources, including teledentistry for the underserved populations, requires further investigation.

## Limitations

Our study has several limitations, including low level of feedback from member participants, small sample size, and limited representation for underserved populations. The study results were presented to the community organizations, and all staff collaborators agreed with the findings and did not provide additional feedback. The 1 participant who provided feedback on the analysis of their personal transcript agreed with the author’s interpretations of their transcript and provided clarification regarding the number of members they witnessed who expressed intent on performing self-extractions before compared to during the pandemic. The low level of feedback on the study results from participants may be due to several factors, including the long delay between the interviews and the offerings to provide feedback, the voluntary aspect of feedback participation as we specifically requested clarification from the 1 participant who did provide feedback, being unable to contact a few member participants, and the possible trauma or difficult experiences of the participants during the pandemic that they would not want to revisit. For greater participation of underserved populations in member checking, it may be ideal to ask participants during their interview if they would like the opportunity to review their interview transcript and request their contact information to involve them in that process.

The findings in this study were derived from a small purposive sample composed of 31 staff and members from 6 organizations in British Columbia, Canada, thereby limiting any generalization. The experiences and perspectives of other underserved populations such as Indigenous communities and people living in rural areas were not captured. As the inclusion criteria included those able to communicate in English, other underserved populations such as newcomers and refugees may not have been represented in this study. In addition, the remote delivery method of the interviews may have decreased rapport with the participants and may have led to missing nonverbal information compared to if the interviews were done in person (Krouwel et al. 2019).

## Conclusion

Underserved populations who already face barriers to oral health care services experienced increased difficulty in addressing their oral health needs and concerns during the beginning of the COVID-19 pandemic. Strategies aimed at reaching out to this population and those who support them are needed to help mitigate negative coping strategies and increased oral health disparities. Further investigation into how to reach underserved populations through various forms of virtual applications is warranted.

## Author Contributions

V. Johnson, contributed to the data acquisition, analysis, and interpretation, drafted and critically revised the manuscript; M. Brondani, contributed to conception, design, and interpretation, critically revised the manuscript; H. von Bergmann, S. Grossman, contributed to design, critically revised the manuscript; L. Donnelly, contributed to conception, design, data acquisition, analysis, and interpretation, critically revised the manuscript. All authors gave final approval and agree to be accountable for all aspects of the work.

## Supplemental Material

sj-docx-1-jct-10.1177_23800844221083965 – Supplemental material for Dental Service and Resource Needs during COVID-19 among Underserved Populations Click here for additional data file. Supplemental material, sj-docx-1-jct-10.1177_23800844221083965 for Dental Service and Resource Needs during COVID-19 among Underserved Populations by V. Johnson, M. Brondani, H. von Bergmann, S. Grossman and L. Donnelly in JDR Clinical & Translational Research
